# Influenza‐associated mortality in Yancheng, China, 2011‐15

**DOI:** 10.1111/irv.12487

**Published:** 2017-11-28

**Authors:** Hongjun Zhang, Qian Xiong, Peng Wu, Yuyun Chen, Nancy H. L. Leung, Benjamin J. Cowling

**Affiliations:** ^1^ Yancheng Center for Disease Prevention and Control Yancheng China; ^2^ WHO Collaborating Centre for Infectious Disease Epidemiology and Control School of Public Health, Li Ka Shing Faculty of Medicine The University of Hong Kong Hong Kong Special Administrative Region Hong Kong China

**Keywords:** burden, China, influenza, mortality, public health

## Abstract

**Introduction:**

The Yangtze river delta in eastern China, centered on Shanghai, is one of the most populated regions of the world with more than 100 million residents. We examined the impact of influenza on excess mortality in Yancheng, a prefecture‐level city with 8.2 million population located 250 km north of Shanghai, during 2011‐2015.

**Methods:**

We obtained individual data on deaths by date, age, sex, and cause in Yancheng from the Chinese Centers for Disease Control and Prevention, and used these to derive weekly rates of mortality from respiratory causes, respiratory and cardiovascular causes combined, and all causes. We used data on influenza‐like illnesses and laboratory detections of influenza to construct a proxy measure of the weekly incidence of influenza virus infections in the community. We used regression models to estimate the association of influenza activity with mortality and excess mortality by age, cause, and influenza type/subtype.

**Results:**

We estimated that an annual average of 4.59 (95% confidence interval: 3.94, 7.41) excess respiratory deaths per 100 000 persons were associated with influenza, which was 4.6% of all respiratory deaths in the years studied. Almost all influenza‐associated excess deaths occurred in persons ≥65 years. Influenza A(H3N2) had the greatest impact on mortality and was associated with around 50% of the influenza‐associated respiratory deaths in the 5 years studied.

**Conclusions:**

Influenza has a substantial impact on respiratory mortality in Yancheng, mainly in older adults. Influenza vaccination has the potential to reduce disease burden, and cost‐effectiveness analysis could be used to compare policy options.

## 1 INTRODUCTION

Influenza virus infections cause substantial morbidity and mortality in annual winter epidemics in temperate locations.^1^ Most influenza virus infections are never laboratory‐confirmed, including even those that result in hospitalization or death.^2^ Therefore, the preferred approach to quantify the mortality impact of influenza epidemics is statistical modeling of time series of mortality rates.^3^ Many studies examine the impact of influenza on deaths with respiratory codes.^3^, ^4^ However, because death coding practices can vary by location, and because influenza can cause deaths that are not coded as respiratory,^3^ it can be informative to include cardiovascular deaths,^5^ and also to examine all‐cause mortality.^3^, ^6^

**Figure 1:**
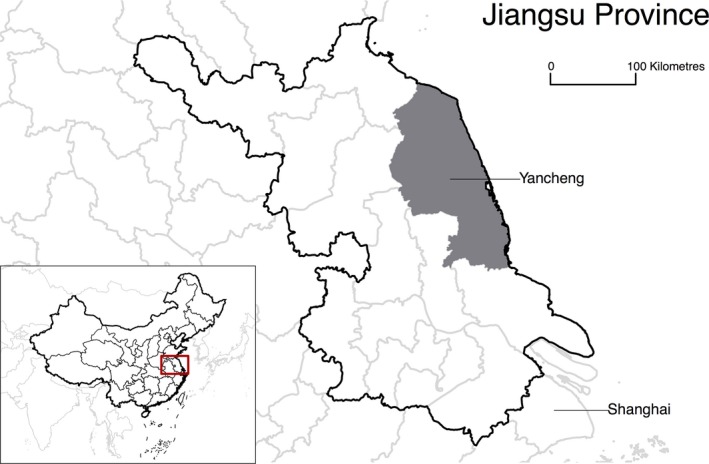
Location of Yancheng in eastern China, just to the north of Shanghai

China is the most populous country in the world with a population of 1.4 billion, and there are now more than 100 cities in China with a population between 1 and 10 million, in addition to the six megacities of Shanghai, Beijing, Tianjin, Guangzhou, Shenzhen and Chengdu. Yancheng is one such city with a population of 7.3 million in the 2010 census, and a gross domestic product of 58 300 RMB (8750 USD) per capita in 2015. Yancheng is situated in Jiangsu province, just north of the Yangtze river delta in eastern China where more than 100 million people are clustered in the area surrounding Shanghai (Figure 1). Jiangsu province has a subtropical‐like pattern in influenza virus activity with a less obvious seasonal pattern, compared to the temperate northern part of the country where there are influenza epidemics each winter, and the southern provinces where there are summer epidemics.^7^ Limited attention has so far been given to the potential burden of influenza in Yancheng, as with most other cities in China, and influenza vaccine coverage is very low. We conducted this study to estimate the influenza‐associated excess mortality burden in Yancheng city in the years 2011 through 2015.

## 2 METHODS

### 2.1 Sources of data

We obtained individual data on deaths by date, age, sex and cause from the Administrative System of Death Records, which form part of the Information System of the Chinese Centers for Disease Control and Prevention. The 10th revision of the International Statistical Classification of Diseases and Related Health Problems (ICD‐10) was used to code the causes of death. We compiled the weekly numbers of deaths from respiratory causes (ICD‐10 codes J00‐J99), cardiovascular and respiratory causes combined (ICD‐10 codes I00‐I99 and J00‐J99), and all causes, by three age groups: 0‐14 years, 15‐64 years, and ≥65 years. Data on the underlying population structure by age and sex from 2011 to 2015 were obtained from the Yancheng Bureau of Statistics, allowing us to derive weekly mortality rates. Influenza surveillance data were collected by the Chinese Influenza Surveillance Information System, a national system which includes sentinel sites in Yancheng, including the weekly rate of influenza‐like illnesses in sentinel hospitals and the weekly proportion of laboratory specimens testing positive for influenza by type/subtype in Yancheng. We multiplied together the weekly influenza‐like illness rate and the weekly influenza detection rates to obtain proxy measures of the weekly incidence rate of influenza virus infections in the community for each type and subtype.^8^, ^9^ Because of relatively small numbers of detections, we smoothed this time series using a moving average with a bandwidth of 5 weeks (Figure S1 in Appendix S1). Data on weekly humidity and temperature were obtained from the China Meteorological Data Service Center.

### 2.2 Statistical methods

We assumed that the influenza activity proxy was a linear correlate of the incidence of infections, and we consequently assumed that there would be a linear association between weekly influenza activity and weekly influenza‐associated mortality.^3^, ^4^ Based on these assumptions, we constructed linear regression models for the mortality rates, including the influenza incidence proxies as covariates and an identity link between the mean mortality rate and the effects of covariates.^3^, ^4^, ^10^, ^11^, ^12^, ^13^ The smoothed virus activity data were used in the main analysis for estimation of excess mortality, and the original virus data were also applied to the same model in a sensitivity analysis for comparison. The models also included temperature and absolute humidity. Because of the average delay between infections and consequent mortality, we lagged the incidence proxy by 2 weeks^14^ and examined the results with 0‐week and 1‐week lags in sensitivity analyses. Excess deaths were estimated by comparing the predicted mortality rates under the model with activity proxies set to zero with the predicted rates with activity proxies set to their observed values.^3^, ^4^ We estimated excess influenza‐associated mortality overall and by age, cause‐of‐death groupings, and influenza type/subtype. The 95% confidence intervals (CIs) for excess mortality rates were estimated with a bootstrap approach.^3^, ^4^ To permit comparison with other countries, excess mortality rates were directly standardized to the World Standard Population.^15^ More details of the statistical model are available in the Appendix S1. Statistical analyses were conducted in R version 3.2.3 (R Foundation for Statistical Computing, Vienna, Austria).

## 3 RESULTS

**Figure 2:**
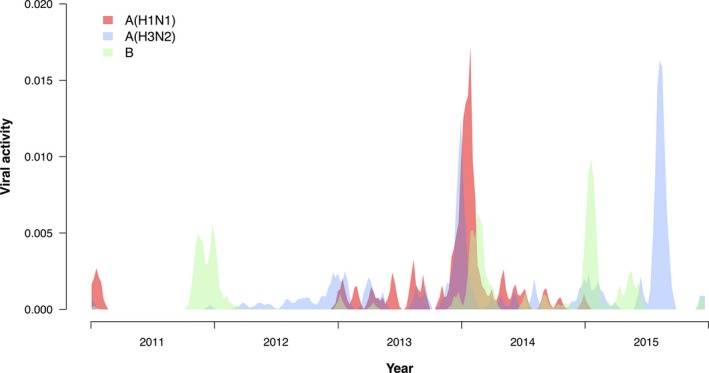
Influenza activity by influenza type/subtype in Yancheng, 2011‐15. We measured weekly influenza activity by multiplying together the weekly rate of influenza‐like illnesses in sentinel surveillance locations with the weekly proportion of laboratory specimens testing positive for influenza by type/subtype. The resulting indicator of influenza activity is assumed to be a correlate of the incidence of influenza virus infections in the population. The plot here shows the smoothed data that were used in the regression models

**Figure 3:**
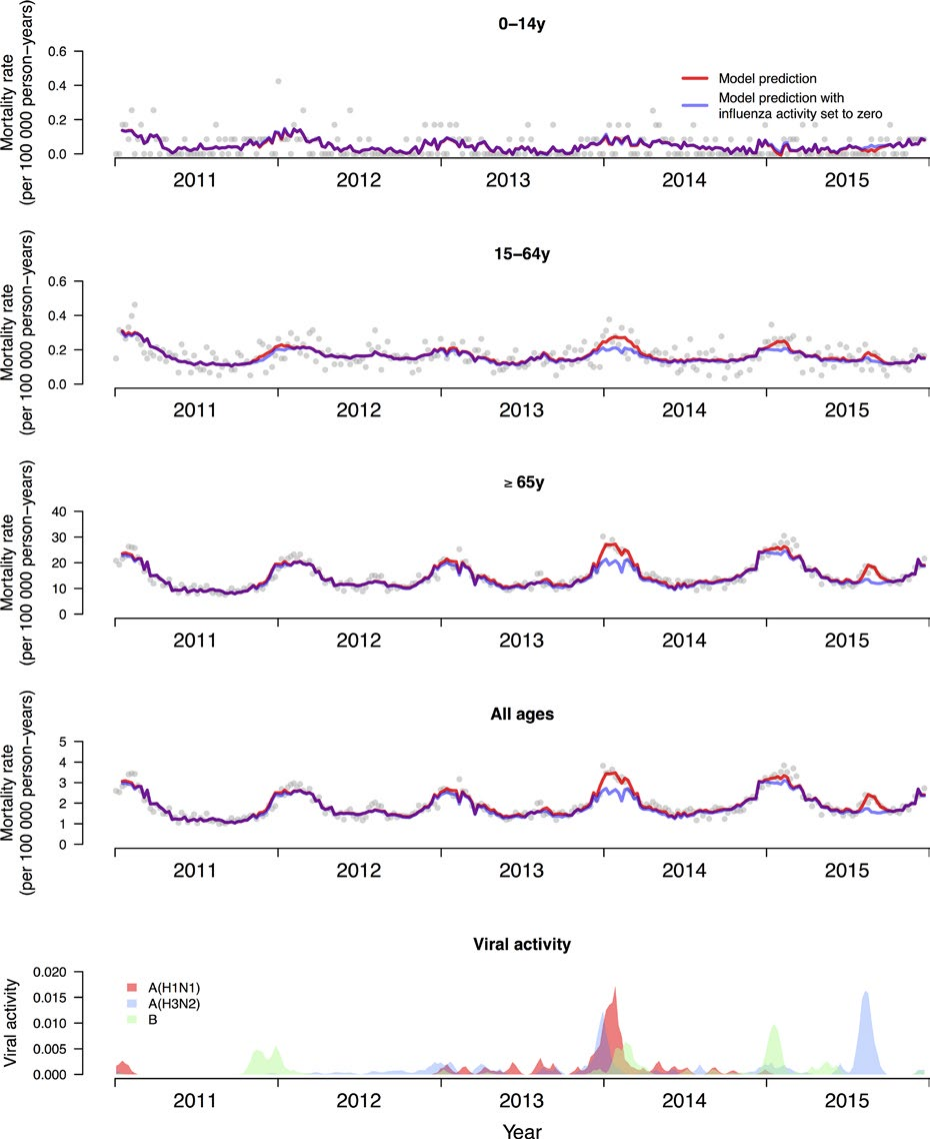
Observed weekly respiratory mortality rates (dots) and modeled rates with influenza activity (red) and with influenza activity set to zero (blue), in three age groups and in all ages. The difference between the red and blue lines was used to estimate the influenza‐associated respiratory mortality. The lowest panel shows the influenza activity proxy

In the 5 years studied, a total of 266 873 deaths were recorded, including 41 207 coded as respiratory deaths, and 132 166 as cardiovascular or respiratory deaths. Influenza epidemics occurred in most winters and in the summer of 2015 (Figure 2). We fitted regression models in each age group and overall, and these models were able to capture patterns in respiratory mortality (Figure 3). We estimated that an annual average of 4.59 (95% confidence interval, CI: 3.94, 7.41) excess respiratory deaths per 100 000 persons were associated with influenza, almost all of which occurred in older adults ≥65 years of age (Table 1). This corresponded to an annual average of 378 (95% CI: 325, 610) respiratory deaths and 572 (95% CI: 235, 1176) all‐cause deaths in all ages in Yancheng. The influenza‐associated respiratory deaths corresponded to 4.6% of all respiratory deaths, and the influenza‐associated all‐cause deaths corresponded to 1.1% of all deaths during the study period. Among older adults ≥65 years of age, our estimates corresponded to 4.6% of respiratory deaths and 1.2% of all deaths. When standardizing the age‐specific excess mortality rates to the World Standard Population, the age‐standardized excess influenza‐associated respiratory mortality rate was 3.21 (95% CI: 1.67, 4.77) per 100 000 persons.

**Table 1:** Estimates of the influenza‐associated annual excess mortality rates by age and by cause of death in Yancheng, 2011‐2015

Cause of death	Average influenza‐associated mortality rate (per 100 000 population per year)							
	0‐14y	(95% CI)	15‐64y	(95% CI)	≥65y	(95% CI)	All ages	(95% CI)
Respiratory diseases	−0.06	(−0.39, 0.22)	0.46	(0.26, 0.90)	35.63	(30.53, 57.34)	4.59	(3.94, 7.41)
Cardiovascular and respiratory diseases	−0.08	(−0.47, 0.27)	1.29	(0.74, 2.63)	45.86	(26.36, 89.68)	6.43	(4.06, 12.31)
All causes	0.52	(−1.06, 2.07)	0.74	(−0.80, 2.73)	49.57	(19.80, 102.50)	6.94	(2.85, 14.28)

CI, confidence interval.

In general, there was an increase in estimated excess influenza‐associated mortality with broader cause‐of‐death groupings (Table 1), except in children 0‐14 years of age where there was negligible excess mortality. When broken down by influenza type/subtype, influenza A(H3N2) had the greatest impact during our study period with an average of 2.31 (95% CI: 1.66, 3.83) excess respiratory deaths per 100 000 persons (Table 2), and 3.44 (95% CI: 0.74, 7.32) excess all‐cause deaths per 100 000 persons (Table 3). This was around 50% (2.31/4.59) of all influenza‐associated respiratory deaths. Influenza A(H3N2) predominated in most of the 5 years studied, including 2012, 2013, 2014, and 2015, and influenza had generally greater impact in the final 3 years studied (Table 4).

**Table 2:** Estimates of excess influenza‐associated respiratory mortality rates by age and by influenza type/subtype in Yancheng, 2011‐2015

Influenza type/subtype	Average influenza‐associated mortality rate (per 100 000 population per year)							
	0‐14y	(95% CI)	15‐64y	(95% CI)	≥65y	(95% CI)	All ages	(95% CI)
Influenza A(H1N1)	0.05	(−0.09, 0.25)	0.14	(0.01, 0.37)	12.25	(7.47, 23.58)	1.58	(0.96, 3.04)
Influenza A(H3N2)	−0.08	(−0.27, 0.10)	0.11	(−0.05, 0.33)	18.68	(14.22, 31.05)	2.31	(1.66, 3.83)
Influenza B	−0.04	(−0.25, 0.15)	0.21	(0.05, 0.45)	4.70	(−2.59, 15.07)	0.71	(−0.23, 2.06)
All influenza	−0.06	(−0.39, 0.22)	0.46	(0.26, 0.90)	35.63	(30.53, 57.34)	4.59	(3.94, 7.41)

CI, confidence interval.

**Table 3:** Estimates of excess influenza‐associated all‐cause mortality rates by age and by influenza type/subtype in Yancheng, 2011‐2015

Influenza type/subtype	Average influenza‐associated mortality rate (per 100 000 population per year)							
	0‐14y	(95% CI)	15‐64y	(95% CI)	≥65y	(95% CI)	All ages	(95% CI)
Influenza A(H1N1)	0.70	(−0.06, 1.88)	−0.05	(−1.05, 1.01)	9.74	(−10.03, 38.23)	1.55	(−1.09, 5.59)
Influenza A(H3N2)	−0.12	(−1.11, 0.75)	0.24	(−0.76, 1.40)	27.02	(8.72, 55.47)	3.44	(0.74, 7.32)
Influenza B	−0.07	(−1.16, 0.82)	0.54	(−0.60, 1.72)	12.81	(−8.53, 43.10)	1.95	(−1.06, 6.06)
All influenza	0.52	(−1.06, 2.07)	0.74	(−0.80, 2.73)	49.57	(19.80, 102.50)	6.94	(2.85, 14.28)

CI, confidence interval.

**Table 4:** Influenza‐associated excess respiratory mortality risk in each year in Yancheng, 2011‐2015, by influenza type/subtype

Year	Predominant strain(s)	Excess mortality risk (per 100 000 population)							
		A(H1N1)pdm09	(95% CI)	A(H3N2)	(95% CI)	B	(95% CI)	All influenza	(95% CI)
2011	B, A(H1N1)pdm09	0.45	(0.17, 0.73)	0.09	(0.05, 0.14)	0.75	(−0.37, 1.98)	1.29	(0.11, 2.56)
2012	A(H3N2)	0.07	(0.03, 0.12)	1.26	(0.71, 1.89)	0.29	(−0.14, 0.76)	1.62	(0.92, 2.43)
2013	A(H3N2), A(H1N1)pdm09	2.88	(1.07, 4.73)	3.32	(1.87, 4.99)	0.13	(−0.07, 0.35)	6.34	(4.22, 8.46)
2014	A(H1N1)pdm09, A(H3N2)	4.45	(1.65, 7.29)	1.90	(1.07, 2.84)	1.19	(−0.59, 3.13)	7.53	(4.29, 10.68)
2015	A(H3N2)	0.03	(0.01, 0.05)	5.01	(2.82, 7.51)	1.22	(−0.61, 3.21)	6.25	(3.38, 9.69)

CI, confidence interval.

In sensitivity analyses, the average annual influenza‐associated respiratory excess mortality was estimated to be 3.10 (95% CI: 2.06, 4.28) per 100 000 persons from the model with the original virus data, largely similar to the main analysis with the smoothed virus data (Tables S4, S5 and S6 in Appendix S1). The main estimates were also generally similar to those from the models with a lag of 1 week and 0 week; that is, influenza was associated with 4.58 (95% CI: 3.70, 7.47) and 4.34 (95% CI: 2.99, 7.98) excess respiratory deaths per 100 000 persons, respectively (Tables S1a‐b, S2a‐b and S3a‐b in Appendix S1).

## 4 DISCUSSION

We estimated an average of 4.59 (95% CI: 3.94, 7.41) excess respiratory deaths per 100 000 persons in Yancheng (Table 1), which is quite consistent with estimates for the impact of influenza across the whole of China,^2^ Hong Kong,^4^, ^16^ and other locations.^6^, ^10^, ^17^ This corresponded to 4.6% of all respiratory deaths during the study period, again consistent with what has been reported elsewhere, for example, 6.0% in Hong Kong,^4^ 2% in the United States,^17^ 3.2% in Australia,^18^ and 4.1% in Bangkok.^10^ Variation in this fraction would be affected to some degree by differences in coding practices between locations.^6^ The impact of influenza was estimated to be even greater when incorporating the impact on cardiovascular deaths and all‐cause mortality (Table 1).

The majority of excess deaths were estimated to occur in older adults (Table 1). This is consistent with the findings of other studies;^6^ for example, 95%, 86%, and 94% of influenza‐associated excess respiratory mortality were estimated to occur in this group in studies in Hong Kong,^4^ China,^2^ and the United States, respectively.^17^ Influenza vaccination is available through the private market in Yancheng, and vaccination coverage is currently very low in all age groups. Our results indicate the potential for influenza vaccination to reduce the mortality impact of influenza particularly in older adults. Cost‐effectiveness analysis could be used to compare policy options.

We identified a greater impact of influenza A(H3N2) compared to influenza A(H1N1) and influenza B (Table 2, Table 3), which is similar to most other locations.^3^, ^4^, ^6^, ^17^, ^19^, ^20^ However, this observation is somewhat different to a previous study that estimated influenza B had a greater impact than influenza A(H3N2) in China, in the years 2003‐08.^2^ We note that the impact of each influenza type/subtype would depend to some extent in patterns in circulation; our study included 5 years of data with variable circulation of A(H1N1), A(H3N2), and B, and two major influenza A(H3N2) epidemics in 2013‐14 and 2015 (Figure 2).

In this study, respiratory excess deaths accounted for most of the influenza‐associated mortality in Yancheng, while the estimated cardiovascular excess deaths were less than half of the respiratory excess deaths. A large variation was observed in the relative contribution of respiratory diseases or cardiovascular diseases to the overall influenza‐associated excess mortality in different studies.^3^, ^4^, ^6^, ^19^ Further investigations may be needed to explore the potential reasons accounting for differences in the estimates across studies.^6^

There are a few limitations of our study. First, our results are dependent on the coding and registration of deaths, and errors in attribution of cause of death, which were used as the outcome time series in our analyses. We did examine all‐cause mortality as a reference point for the estimated effects on respiratory mortality and respiratory and cardiovascular mortality. Second, our results are also dependent on the measure of the incidence of influenza virus infections in the underlying population, which we based on clinical and laboratory surveillance data (Figure 2). While we were aware that Poisson family models are conventionally used for statistical modeling of count data and incidence rates,^21^ we considered the mechanism by which influenza incidence leads to deaths and hypothesized that there should be a linear correlation between incidence and mortality.^3^ We therefore used an identity link rather than a logarithmic link to relate incidence to mortality in our model, as many others have done.^3^, ^4^, ^6^, ^10^, ^11^, ^12^, ^13^ We used a normal error distribution, but results would have been similar with a Poisson family error distribution as the numbers of deaths each week were not small. Finally, we only examined the impact of influenza epidemics on excess mortality. Influenza epidemics also cause a substantial health impact on hospitalizations and outpatient medical consultations, and consequent economic impact including healthcare costs and lost productivity. A more comprehensive assessment of the impact of influenza could further inform public health strategies including the use of vaccination.

In conclusion, we documented a substantial burden of influenza in Yancheng, and it is likely that a similar burden occurs in other cities in eastern China where vaccination coverage is very low. These estimates of influenza‐associated mortality can be used to guide public health policy decisions. Our results highlight the important burden of influenza virus in older adults and the potential for influenza vaccination to reduce disease burden.

## CONFLICT OF INTEREST

BJC reports receipt of research funding from Sanofi Pasteur for a study of influenza vaccine effectiveness. The authors report no other potential conflict of interests.
