# Supplementary material for: Influenza‐associated mortality in Yancheng, China, 2011‐15
Source: Influenza Other Respir Viruses. 2017 Nov 28;12(1):98–103. doi: 10.1111/irv.12487 (PMC5818359; doi:10.1111/irv.12487)
Supplement: Supplementary file 1 [file IRV-12-98-s001.docx]

Supplementary Information

**Statistical model**

We used a linear regression model to estimate the association between the weekly death rates and all covariates, the regression equation is as follows:

$$E\left( \frac{D_{i}}{{pop}_{i}^{w}} \right)=n\left( T_{i-2}, {df}_{T} \right)+n\left( H_{i-2}, {df}_{H} \right)+{H1N1}_{i-2}*{ILI}_{i-2}+{H3N2}_{i-2}*{ILI}_{i-2}+B_{i-2}*{ILI}_{i-2} + n(i-2, {df}_{w})$$

In above equation, $E\left( \frac{D_{i}}{{pop}_{i}^{w}} \right)$ means the expected mortality rate in week *i*. ${pop}_{i}^{w}$ means population number in week *i.* $T_{i-2}$ and $H_{i-2}$ denote the effects of temperature and absolute humidity on deaths in week *i* -2, respectively. ${H1N1}_{i-2}$, ${H3N2}_{i-2}$, and $B_{i-2}$ represent the weekly viral activity of different influenza subtypes in week *i* -2. ${ILI}_{i-2}$ is defined as the proportion of consultations attributable to influenza-like-illness (ILI) from sentinel general practitioners. The product of proportion of viral isolates and proportion of consultations due to ILI was used as the proxy of the virus activity. $n\left( T_{i-2}, {df}_{T} \right)$, $n\left( H_{i-2}, {df}_{H} \right)$, and $n(i-2, {df}_{w})$ denote the cubic smooth splines of the trends in the weekly temperature, weekly humidity and weekly calendar time, with the degree of freedom ${df}_{T}$, ${df}_{H}$ and ${df}_{w}$, respectively. Errors were assumed to follow a normal distribution.

In our primary model, we assumed that there was a two-week lag between the time-varying covariates and mortality by using smoothing surveillance data. We conducted sensitivity analyses to examine the impact of the assumed time lag on the estimated influenza-associated excess mortality with 1 and 0 week lag by using smoothed surveillance data. The results are shown in Table S1a-b, Table S2a-b and Table S3a-b. We also conducted sensitivity analyses to obtain the influenza of the original data with lag 2 weeks, the results are shown in Table S4, Table S5 and Table S6.

**Smoothing of the viral data**

We used a 5-week moving average to smooth the viral data. Therefore, the smoothed weekly virus activity data ${H1N1}_{i}$, ${H3N2}_{i}$ and $B_{i}$ would be obtained by following equation:

$y_{s}\left( 1 \right)=y(1)$ $(i=1)$

$y_{s}\left( 2 \right)=(y\left( 1 \right)+y\left( 2 \right)+y(3))/3$ $(i=2)$

$y_{s}\left( i \right)=\frac{1}{5}(y\left( i+2 \right)+y\left( i+1 \right)+ y+y\left( i-1 \right)+y\left( i-2 \right))$ $(i \geq3)$

Where i is the week number.

The patterns of virus activity before and after smoothing are shown in Figure S1.

Figure S1. Patterns of virus activity before and after smoothing

Table S1a. Estimates of excess influenza-associated mortality rates by age and by cause of death in Yancheng, 2011-2015 (lag one week)

| Cause of death | Average influenza-associated mortality rate (per 100,000 population per year) | | | | | | | |
| --- | --- | --- | --- | --- | --- | --- | --- | --- |
|  | 0-14y | (95% CI) | 15-64y | (95% CI) | ≥65y | (95% CI) | All ages | (95% CI) |
| Respiratory diseases | -0.06 | (-0.38, 0.29) | 0.46 | (0.26, 0.86) | 35.45 | (27.86, 58.08) | 4.58 | (3.70, 7.47) |
| Cardiovascular & respiratory diseases | 0.13 | (-0.21, 0.53) | 1.27 | (0.60, 2.47) | 42.04 | (18.67, 83.24) | 5.98 | (3.00, 11.60) |
| All causes | 0.27 | (-1.34, 2.14) | 0.08 | (-1.80, 1.88) | 44.15 | (10.68, 95.88) | 5.71 | (1.12, 12.57) |

CI: confidence interval

Table S1b. Estimates of excess influenza-associated mortality rates by age and by cause of death in Yancheng, 2011-2015 (lag 0 week)

| Cause of death | Average influenza-associated mortality rate (per 100,000 population per year) | | | | | | | |
| --- | --- | --- | --- | --- | --- | --- | --- | --- |
|  | 0-14y | (95% CI) | 15-64y | (95% CI) | ≥65y | (95% CI) | All ages | (95% CI) |
| Respiratory diseases | -0.33 | (-0.68, -0.06) | 0.39 | (0.188, 0.80) | 34.28 | (22.79, 63.07) | 4.34 | (2.99, 7.98) |
| Cardiovascular & respiratory diseases | -0.06 | (-0.40, 0.29) | 0.94 | (0.25, 2.17) | 38.5 | (-1.98, 93.36) | 5.29 | (0.32, 12.67) |
| All causes | -0.04 | (-1.68, 1.65) | -0.71 | (-2.66, 1.01) | 46.04 | (-6.03, 119.20) | 5.20 | (-1.80, 14.58) |

CI: confidence interval

Table S2a. Estimates of excess influenza-associated respiratory mortality rates by age and by influenza type/subtype in Yancheng, 2011-2015 (lag one week)

| Influenza type/subtype | Average influenza-associated mortality rate (per 100,000 population per year) | | | | | | | |
| --- | --- | --- | --- | --- | --- | --- | --- | --- |
|  | 0-14y | (95% CI) | 15-64y | (95% CI) | ≥65y | (95% CI) | All ages | (95% CI) |
| Influenza A(H1N1) | 0.11 | (-0.04, 0.35) | 0.19 | (0.05, 0.41) | 16.2 | (10.16, 29.11) | 2.09 | (1.35, 3.70) |
| Influenza A(H3N2) | -0.09 | (-0.30, 0.08) | 0.11 | (-0.05, 0.29) | 17.02 | (10.66, 28.59) | 2.1 | (1.30, 3.53) |
| Influenza B | -0.08 | (-0.28, 0.14) | 0.17 | (0.02, 0.40) | 2.23 | (-6.66, 13.32) | 0.38 | (-0.77, 1.74) |
| All influenza | -0.06 | (-0.38, 0.29) | 0.46 | (0.26, 0.86) | 35.45 | (27.86, 58.08) | 4.58 | (3.70, 7.47) |

CI: confidence interval

Table S2b. Estimates of excess influenza-associated respiratory mortality rates by age and by influenza type/subtype in Yancheng, 2011-2015 (lag 0 week)

| Influenza type/subtype | Average influenza-associated mortality rate (per 100,000 population per year) | | | | | | | |
| --- | --- | --- | --- | --- | --- | --- | --- | --- |
|  | 0-14y | (95% CI) | 15-64y | (95% CI) | ≥65y | (95% CI) | All ages | (95% CI) |
| Influenza A(H1N1) | 0.02 | (-0.17, 0.21) | 0.24 | (0.06, 0.43) | 22.67 | (8.95, 35.05) | 2.89 | (1.19, 4.35) |
| Influenza A(H3N2) | -0.18 | (-0.36, 0.01) | 0.004 | (-0.16, 0.19) | 10.75 | (-0.69, 23.02) | 1.26 | (-0.19, 2.74) |
| Influenza B | -0.16 | (-0.35, 0.08) | 0.14 | (-0.05, 0.35) | 0.74 | (-12.89, 15.65) | 0.17 | (-1.55, 2.00) |
| All influenza | -0.33 | (-0.68, -0.06) | 0.39 | (0.188, 0.80) | 34.28 | (22.79, 63.07) | 4.34 | (2.99, 7.98) |

CI: confidence interval

Table S3a. Estimates of excess influenza-associated all-cause mortality rates by age and by influenza type/subtype in Yancheng, 2011-2015 (lag one week)

| Influenza type/subtype | Average influenza-associated mortality rate (per 100,000 population per year) | | | | | | | |
| --- | --- | --- | --- | --- | --- | --- | --- | --- |
|  | 0-14y | (95% CI) | 15-64y | (95% CI) | ≥65y | (95% CI) | All ages | (95% CI) |
| Influenza A(H1N1) | 0.33 | (-0.57, 1.41) | -0.01 | (-1.19, 0.91) | 8.72 | (-14.99, 34.27) | 1.4 | (-1.90, 4.96) |
| Influenza A(H3N2) | -0.07 | (-0.95, 0.88) | 0.27 | (-0.77, 1.39) | 32.61 | (14.14, 65.31) | 4.07 | (1.15, 8.43) |
| Influenza B | 0.01 | (-1.03, 1.11) | -0.18 | (-1.29, 1.05) | 2.82 | (-25.21, 34.39) | 0.24 | (-3.72, 4.41) |
| All influenza | 0.27 | (-1.34, 2.14) | 0.083 | (-1.80, 1.88) | 44.15 | (10.68, 95.88) | 5.71 | (1.12, 12.57) |

CI: confidence interval

Table S3b. Estimates of excess influenza-associated all-cause mortality rates by age and by influenza type/subtype in Yancheng, 2011-2015 (lag 0 week).

| Influenza type/subtype | Average influenza-associated mortality rate (per 100,000 population per year) | | | | | | | |
| --- | --- | --- | --- | --- | --- | --- | --- | --- |
|  | 0-14y | (95% CI) | 15-64y | (95% CI) | ≥65y | (95% CI) | All ages | (95% CI) |
| Influenza A(H1N1) | 0.14 | (-0.70, 1.21) | -0.13 | (-1.24, 0.90) | 17.42 | (-14.28, 56.66) | 2.27 | (-1.85, 7.19) |
| Influenza A(H3N2) | -0.2 | (-1.21, 0.66) | -0.09 | (-1.16, 1.05) | 30.64 | (3.82, 72.96) | 3.48 | (-0.27, 8.66) |
| Influenza B | 0.02 | (-1.02, 1.07) | -0.5 | (-1.80, 0.58) | -2.03 | (-38.82, 37.24) | -0.54 | (-5.31, 4.54) |
| All influenza | -0.04 | (-1.68, 1.65) | -0.71 | (-2.66, 1.01) | 46.04 | (-6.03, 119.20) | 5.20 | (-1.80, 14.58) |

CI: confidence interval

Table S4. Estimates of excess influenza-associated mortality rates by age and by cause of death in Yancheng in 2011-2015 using the original virus surveillance data (lag two weeks).

| Cause of death grouping | Average influenza-associated mortality rate (per 100,000 population per year) | | | | | | | |
| --- | --- | --- | --- | --- | --- | --- | --- | --- |
|  | 0-14y | (95% CI) | 15-64y | (95% CI) | ≥65y | (95% CI) | All ages | (95% CI) |
| Respiratory diseases | -0.04 | (-0.27, 0.17) | 0.36 | (0.15, 0.59) | 23.74 | (15.60, 32.83) | 3.10 | (2.06, 4.28) |
| Cardiovascular & respiratory diseases | -0.08 | (-0.36, 0.19) | 0.73 | (0.20, 1.43) | 35.34 | (17.45, 56.81) | 4.76 | (2.49, 7.64) |
| All causes | 0.35 | (-0.96, 1.36) | 0.88 | (-0.34, 2.13) | 36.50 | (11.40, 63.90) | 5.28 | (1.71, 9.28) |

CI: confidence interval

Table S5. Estimates of excess influenza-associated respiratory mortality rates by age and by influenza type/subtype in Yancheng in 2011-2015 using the original virus surveillance data (lag two weeks).

| Influenza type/subtype | Average influenza-associated mortality rate (per 100,000 population per year) | | | | | | | |
| --- | --- | --- | --- | --- | --- | --- | --- | --- |
|  | 0-14y | (95% CI) | 15-64y | (95% CI) | ≥65y | (95% CI) | All ages | (95% CI) |
| Influenza A(H1N1) | 0.01 | (-0.09, 0.12) | 0.11 | (0.005, 0.22) | 7.95 | (4.04, 12.15) | 1.03 | (0.53, 1.56) |
| Influenza A(H3N2) | -0.005 | (-0.13, 0.13) | 0.11 | (-0.02, 0.25) | 12.51 | (7.18, 18.31) | 1.58 | (0.88, 2.32) |
| Influenza B | -0.05 | (-0.20, 0.11) | 0.14 | (-0.01, 0.29) | 3.28 | (-2.28, 9.57) | 0.49 | (-0.23, 1.26) |
| All influenza | -0.04 | (-0.27, 0.17) | 0.36 | (0.15, 0.59) | 23.74 | (15.60, 32.83) | 3.10 | (2.06, 4.28) |

CI: confidence interval

Table S6. Estimates of excess influenza-associated all-cause mortality rates by age and by influenza type/subtype in Yancheng in 2011-2015 using the original virus surveillance data (lag two weeks).

| Influenza type/subtype | Average influenza-associated mortality rate (per 100,000 population per year) | | | | | | | |
| --- | --- | --- | --- | --- | --- | --- | --- | --- |
|  | 0-14y | (95% CI) | 15-64y | (95% CI) | ≥65y | (95% CI) | All ages | (95% CI) |
| Influenza A(H1N1) | 0.71 | (0.11, 1.25) | 0.16 | (-0.40, 0.80) | 8.60 | (-2.23, 22.34) | 1.37 | (-0.06, 3.32) |
| Influenza A(H3N2) | -0.23 | (-0.97, 0.47) | 0.14 | (-0.59, 0.94) | 18.86 | (3.38, 35.38) | 2.41 | (0.16, 4.75) |
| Influenza B | -0.14 | (-0.96, 0.56) | 0.58 | (-0.37, 1.35) | 9.03 | (-6.82, 27.06) | 1.50 | (-0.78, 3.92) |
| All influenza | 0.35 | (-0.96, 1.36) | 0.88 | (-0.34, 2.13) | 36.5 | (11.40, 63.90) | 5.28 | (1.71, 9.28) |

CI: confidence interval
